# Alicyclic Polyimide/SiO_2_ Mixed Matrix Membranes for Water/n-Butanol Pervaporation

**DOI:** 10.3390/membranes11080564

**Published:** 2021-07-27

**Authors:** Ching-Wen Hsieh, Bo-Xian Li, Shing-Yi Suen

**Affiliations:** 1 Department of Chemical Engineering, National Chung Hsing University, Taichung 402, Taiwan; d100065008@mail.nchu.edu.tw (C.-W.H.); sa4104065009@smail.nchu.edu.tw (B.-X.L.); 2 i-Center for Advanced Science and Technology, National Chung Hsing University, Taichung 402, Taiwan

**Keywords:** mixed matrix membrane, alicyclic polyimide, pervaporation

## Abstract

Alicyclic polyimides (PIs) have excellent properties in solubility, mechanical strength, thermal property, etc. This study developed two types of alicyclic PI-based mixed matrix membranes (MMMs) for water/n-butanol pervaporation application, which have never been investigated previously. The fillers were hydrophilic SiO_2_ nanoparticles. The synthesized PI was mixed with SiO_2_ nanoparticles in DMAc to make the casting solution, and a liquid film was formed over PET substrate using doctor blade. A dense MMM was fabricated at 80 °C and further treated via multi-stage curing (100–170 °C). The prepared membranes were characterized by FTIR, TGA, FE-SEM, water contact angle, and solvent swelling. The trends of pure solvent swelling effects agree well with the water contact angle results. Moreover, the pervaporation efficiencies of alicyclic PI/SiO_2_ MMMs for 85 wt% n-butanol aqueous solution at 40 °C were investigated. The results showed that BCDA-3,4′-ODA/SiO_2_ MMMs had a larger permeation flux and higher separation factor than BCDA-1,3,3-APB/SiO_2_ MMMs. For both types of MMMs, the separation factor increased first and then decreased, with increasing SiO_2_ loading. Based on the PSI performance, the optimal SiO_2_ content was 0.5 wt% for BCDA-3,4′-ODA/SiO_2_ MMMs and 5 wt% for BCDA-1,3,3-APB/SiO_2_ MMMs. The overall separation efficiency of BCDA-3,4′-ODA-based membranes was 10–30-fold higher.

## 1. Introduction

Membrane separation technology is mainly based on the principle of using the membrane as an interfacial barrier to separate the feed into two outlet phases: permeate and retentate. The difference in the rate of molecular permeation through the membrane is the crucial factor required to achieve the separation goal. The driving force could be pressure difference, concentration difference, temperature difference, potential difference, or a combination. Pervaporation is a membrane separation technique associating two mechanisms of permeation and evaporation [1,2]. The liquid mixture is loaded in direct contact with the frontal membrane surface, then passes through the membrane by partial vaporization, and finally leaves the other side of the membrane in vapor form. Ingredient selection and separation are governed via the distinction in solubility and diffusivity throughout the membrane. In addition to efficient separation, pervaporation process provides other benefits such as a simple design, compact space, easy operation and maintenance, low energy consumption, etc., [3,4,5]. These advantages lead to a wide application of areas for pervaporation: organic solvent/water separation [6], the removal of volatile organic compounds (VOCs) from water [7], aromatic/aliphatic hydrocarbon separation [8], dehydration to enhanced esterification [9], dehydration of glycerin solution [10], azeotropic solvent purification [11,12], acetone butanol ethanol (ABE) recovery in fermentation process [13], and so on.

The solution–diffusion model is usually adopted to describe the separation mechanism of pervaporation using a nonporous membrane [14,15]. In the beginning, the components in the liquid feed are sorbed into the membrane due to their affinities with membrane material. A higher affinity results in a higher sorption amount (solubility), while lower-affinity molecules are partially retained. The sorbed molecules diffuse through the membrane and are vaporized. The diffusion rate depends on the size and shape of molecule. The permeating components are removed and collected by either applying a very low pressure (vacuum) or flowing an inert gas (sweeping gas) in the permeate side. The vacuum mode allows the permeants to quickly vaporize and desorb.

To minimize energy consumption and maximize separation efficiency, the membrane material is favorably selected to have a higher affinity with the minor components in the feed [16,17,18,19]. For example, hydrophilic membrane is generally applied for water as the minor component, and vice versa. The popular hydrophilic polymers as the membrane materials for dehydration purposes in the literature [20,21,22,23,24,25,26] include: polyvinyl alcohol (PVA), polyacrylonitrile (PAN), polyimide (PI), sulfonated polybenzimidazole (SPBI), sodium alginate (NaAlg), chitosan (CS), etc. On the other hand, the promising hydrophobic polymeric membranes for the removal of organic compounds contain polydimethylsiloxane (PDMS), poly((3,3,3-trifluoropropyl)methylsiloxane) (PTFPMS), etc., [27,28,29]. Inorganic membranes such as graphene, zeolite, and ceramic (e.g., titania, alumina, zirconia, etc.,) are also employed for pervaporation [30,31,32,33]. However, the production expense of inorganic membranes is usually much higher than the polymeric membranes.

In recent years, increasing attention has been drawn to polymeric membranes incorporating inorganic fillers, well known as mixed matrix membranes (MMMs), for combining both the advantages of polymeric membrane and inorganic filler in order to improve the pervaporation performance. The inorganic fillers tested include zeolite, alumina nanoparticles, graphene oxide (with hydroxyl and epoxy groups), magnesium oxide, metal organic frameworks (MOF), functionalized carbon nanotubes, and so on [34,35,36,37,38,39,40]. Generally, these inorganic fillers could raise the hydrophilicity of the membrane to enhance the water flux for the purpose of dehydration [39,40]. Therefore, a proper choice for base material and filler is essentially required to develop the membrane with suitable properties and reliable stability for pervaporation.

PI has been an excellent potential polymer for versatile applications in the chemical industry. Its synthetic chemical structure and the related separation efficiency could be designed and optimized based on the separation purpose [22]. Moreover, PIs possess admirable thermal and mechanical properties, as well as good stability in most organic solvents. For dehydration pervaporation, aromatic PIs have shown good applicability due to their high selectivity towards water, which is attributed to the favored hydrogen bonding between water molecules and the imide groups, in addition to the low free volume of PI for preferential diffusion of smaller water molecules [41,42]. However, there have been very few reports on the use of alicyclic PI membranes in pervaporation so far. Compared to aromatic PIs, alicyclic PIs display good solubility, a low dielectric constant, and high optical transparency, without sacrificing the mechanical and thermal properties [43,44,45,46,47]. The aim of this study was to develop the water/n-butanol pervaporation application using alicyclic PI-based MMMs.

Water/n-butanol separation becomes an important issue due to the widespread interest in searching for alternative energy sources for fossil fuel depletion and eco-friendly consideration [48,49,50]. Bioalcohols produced from biomass are the most promising gasoline substitute. In comparison with lower-carbon-containing alcohols, n-butanol has a higher potential to reduce the fuel consumption and is a better substance blending into diesel and gasoline fuel, owing to its higher energy content, lower volatility, higher flash point, and less ignition problems [49,50]. For reliable applications, biofuel purity needs to reach a very high value. Subsequently, n-butanol purification is usually performed via two processes in the industries: distillation and pervaporation. The dehydration of n-butanol through pervaporation is indeed a main task at the final stage of purification [49,50]. The alicyclic PIs adopted in this study are BCDA-3,4′-ODA and BCDA-1,3,3-APB, which have never been investigated for pervaporation applications until now. To improve the water/n-butanol separation efficiency, SiO_2_ nanoparticles were selected as the fillers, since SiO_2_ is hydrophilic, easy to obtain, and inexpensive [42]. The pervaporation performances of BCDA-3,4′-ODA/SiO_2_ MMMs and BCDA-1,3,3-APB/SiO_2_ MMMs were systematically explored in this work.

## 2. Materials and Methods

### 2.1. Materials

Bicyclo[2.2.2]oct-7-ene-2,3,5,6-tetracarboxylic dianhydride (BCDA), 3,4′-oxydianiline (3,4′-ODA), and 1,3-bis(3-aminophenoxy)benzene (1,3,3-APB) were purchased from Sigma-Aldrich (St. Louis, MO, USA) and used without further purification. γ-Butyrolactone (GBL) and N,N-dimethylacetamide (DMAc) from Sigma-Aldrich were selected as solvents and dried overnight with molecular sieves prior to use. Colloidal silica solution, which contains ca. 20 wt% of 20 nm SiO_2_ nanoparticles in DMAc, was provided from Nissan Chemical (Tokyo Japan).

### 2.2. Preparation of PIs

**Figure 1:**
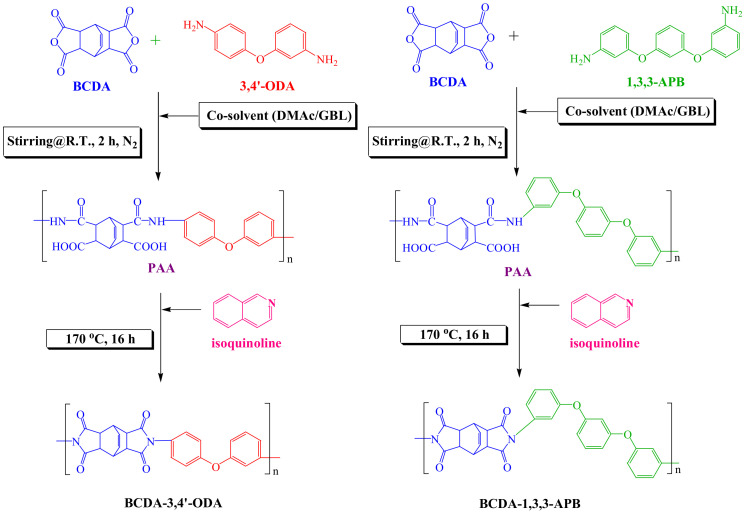
Synthetic routes of alicyclic PIs, BCDA-3,4′-ODA, and BCDA-1,3,3-APB.

The PI synthesis process is depicted in Figure 1. The molar ratio of BCDA-3,4′-ODA or BCDA-1,3,3-APB was 1. The monomers and 4-fold weight of co-solvent (DMAc/GBL = 60/40 (*w*/*w*)) were added to a three-necked flask equipped with a mechanical stirrer, a thermometer, and two connected condensers. The flask was purged with nitrogen, and the mixture was stirred at room temperature for 2 h to react and create a homogeneous poly(amic acid) (PAA) solution. The catalyst isoquinoline (2 wt%) was then added into the three-necked flask, and the temperature was increased to 170 °C. The solution was refluxed at 170 °C for 16 h until the imidization of PAA was completed. The product solution was slowly cooled down, and excess ethanol was poured into the solution for PI precipitation in order to separate it from the unreacted monomers or low-molecular-weight PI. Finally, the PI precipitate was dried in a vacuum oven at 150 °C for 8 h.

### 2.3. Preparation and Characterization of PI/SiO_2_ Mixed Matrix Membranes

20 wt% PI was dissolved in DMAc at room temperature for 2 h. The PI solution was mixed with the colloidal silica solution at a certain ratio for 5 min to make the casting solution. The casting solution was then spread over a clean PET substrate using a 250 µm blade to form a liquid film. The liquid film with the PET substrate was placed in an oven at 80 °C for 1 h to fabricate a dense membrane. The membrane was peeled off from PET and further treated via multi-stage curing. The curing temperature was 100 °C, 140 °C, 150 °C, and 160 °C, and the curing time was 1 h for each stage. In the final stage, the temperature was raised to 170 °C and the membrane was cured for 5 h. The preparation procedures for pristine PI membranes were similar to the above for PI/SiO_2_ MMM, without the blending of SiO_2_ nanoparticles.

The prepared membranes were characterized using a thickness gauge (293–140-30, Mitutoyo, Kanagawa, Japan), FTIR (Cary 630, Agilent, Tokyo, Japan), TGA (Q500, TA Instruments, New Castle, DE, USA), FE-SEM (JSM-6700F, Jeol, Tokyo, Japan), and contact angle (SEO: Phoenix-I portable contact angle analyzer).

### 2.4. Swelling Experiment

The prepared membrane was cut into a size of 3 cm × 3 cm and dried in an oven for 24 h. The membrane weight was recorded as dry weight W_d_ (g). Next, the membrane was soaked into pure alcohol (n-butanol or ethanol) or pure deionized water at 40 °C. After 6 h, the membrane was taken out and the remaining liquid on the membrane surface was wiped. The membrane weight was recorded as wet weight W_s_ (g). The degree of swelling (DS) of the membrane was then calculated with the following equation [51]:

(1) $$DS(\%)=\frac{W_{s}-W_{d}}{W_{d}}\times 100\%.$$

### 2.5. Pervaporation Experiment

**Figure 2:**
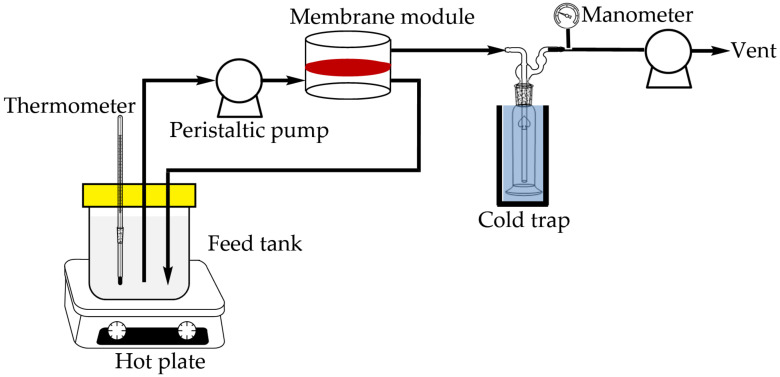
Experimental set-up for pervaporation process.

In the pervaporation process, 1 L of 85 wt% n-butanol aqueous solution at 40 °C was poured into the feed container and pumped into the module with a piece of membrane disc (effective surface area: 19.63 cm^2^). The feed solution passed in the upstream at a flow rate of 9 L/h, and circulated back to the feed container. The permeate was vacuumed and collected in a cold trap. The experimental set-up is illustrated in Figure 2. The duration of pervaporation process was 6 h. The compositions of the solution remained in the feed container and the permeate were analyzed by HPLC. The HPLC system consisted of a pump (600 Controller, Waters), a refractive index detector (RI-101, Shodex), a C8 column (Luna 5 µm C8(2) 100 Å, Phenomenex), and a computer with integration software installed. The mobile phase was pure water, and flow rate was 0.5 mL/min. For each injection, the sample amount was 20 μL.

The total flux permeating through the membrane was calculated as follows:

(2) $$J=\frac{W}{At},$$

where W is the total mass of the permeate (g), A is the effective membrane area (m^2^), and t is the duration time of the experiment (h). Considering that the permeation flux is usually affected by membrane thickness, a normalized permeation flux (J_N_) may be expressed in terms of a certain membrane thickness (L_N_, the average membrane thickness of 30 μm was adopted in this study) for normalization, as given in the following:

(3) $$J_{N}=\frac{JL}{L_{N}},$$

where L is the thickness of the membrane adopted in pervaporation process. The separation factor was determined as:

(4) $$\alpha =\frac{y/(1-y)}{x/(1-x)},$$

where y and x represent the weight fractions of water in the permeate and feed, respectively. In addition, the overall performance of the membrane, combining permeation flux and separation factor together, could be evaluated by pervaporation separation index (PSI) as:

(5) $$PSI=J_{N}(\alpha -1).$$

## 3. Results and Discussion

### 3.1. Membrane Characterization

**Figure 3:**
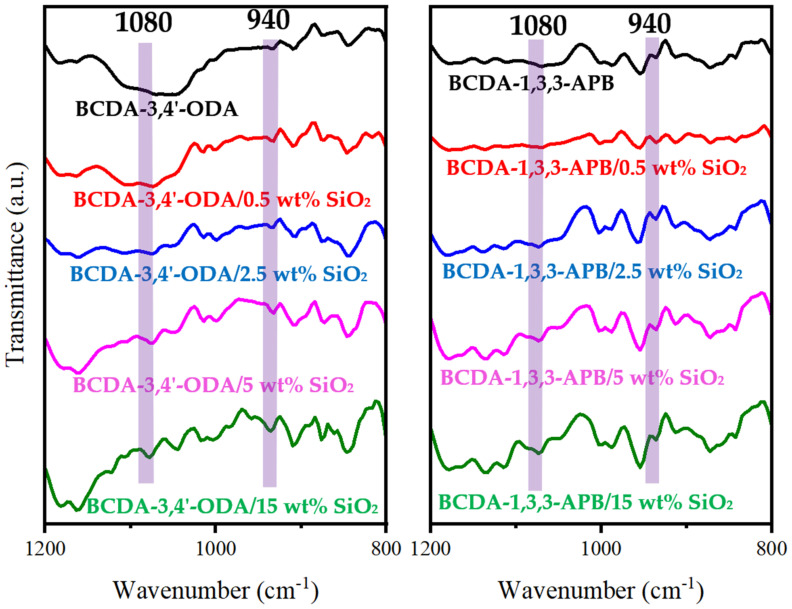
FTIR spectra of pristine alicyclic PI membranes and their related PI/SiO_2_ mixed matrix membranes.

Figure 3 presents the FTIR spectra of pristine alicyclic PI membranes and their related PI/SiO_2_ MMMs. In comparison with the pristine PI membranes, the characteristic peaks of Si-O-Si at 1080 cm^−1^ and Si-O(H) at 940 cm^−1^ [52,53,54] were observed for all the PI/SiO_2_ MMMs. It verified the successful incorporation of SiO_2_ nanoparticles in alicyclic PI-based matrices.

**Figure 4:**
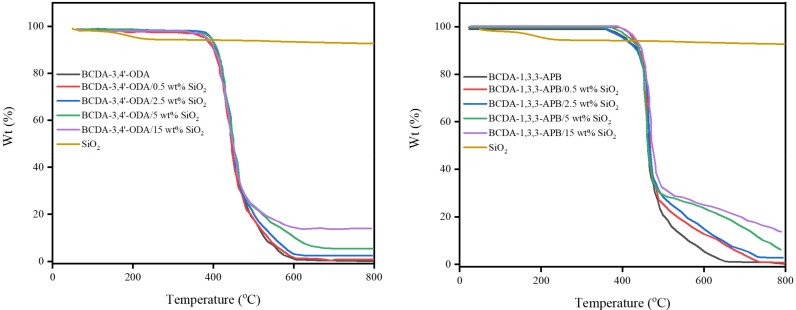
TGA results of pristine alicyclic PI membranes and their related PI/SiO_2_ mixed matrix membranes.

According to the TGA results in Figure 4, both pristine alicyclic PI membranes (BCDA-3,4′-ODA and BCDA-1,3,3-APB) exhibited excellent thermal stability because their temperatures for 5% weight loss (T_5%_) were higher than 350 °C. Compared to pristine alicyclic PI membranes, the TGA curves for PI/SiO_2_ MMMs shifted toward slightly higher temperatures. It implies that the thermal stability of the membrane was enhanced with the incorporation of inorganic SiO_2_ nanoparticles.

The residual wt% of PI/SiO_2_ MMM in Figure 4 was contributed from the undecomposed SiO_2_ nanoparticles, and the value was elevated with the increasing SiO_2_ content. The actual SiO_2_ wt% in PI/SiO_2_ MMM was then calculated from the residual wt% values of the relevant TGA curves as follows:

(6) $$SiO_{2}wt\%=\frac{Y-X}{W-X}\times 100\%,$$

where Y = residual wt% of PI/SiO_2_ MMM, X = residual wt% of pristine PI membrane, W = residual wt% of SiO_2_ particles. The results are listed in Table 1. The actual SiO_2_ contents in PI/SiO_2_ MMMs were a little greater than the values used in the experiments of the membrane preparation. It is possible that some polymer was vanished with solvent vaporization during the curing stage so that the resulted inorganic filler content in MMM was increased. In the subsequent text of this paper, the values of SiO_2_ wt% adopted in the experiment are presented, since the actual contents are close to them.

**Figure 5:**
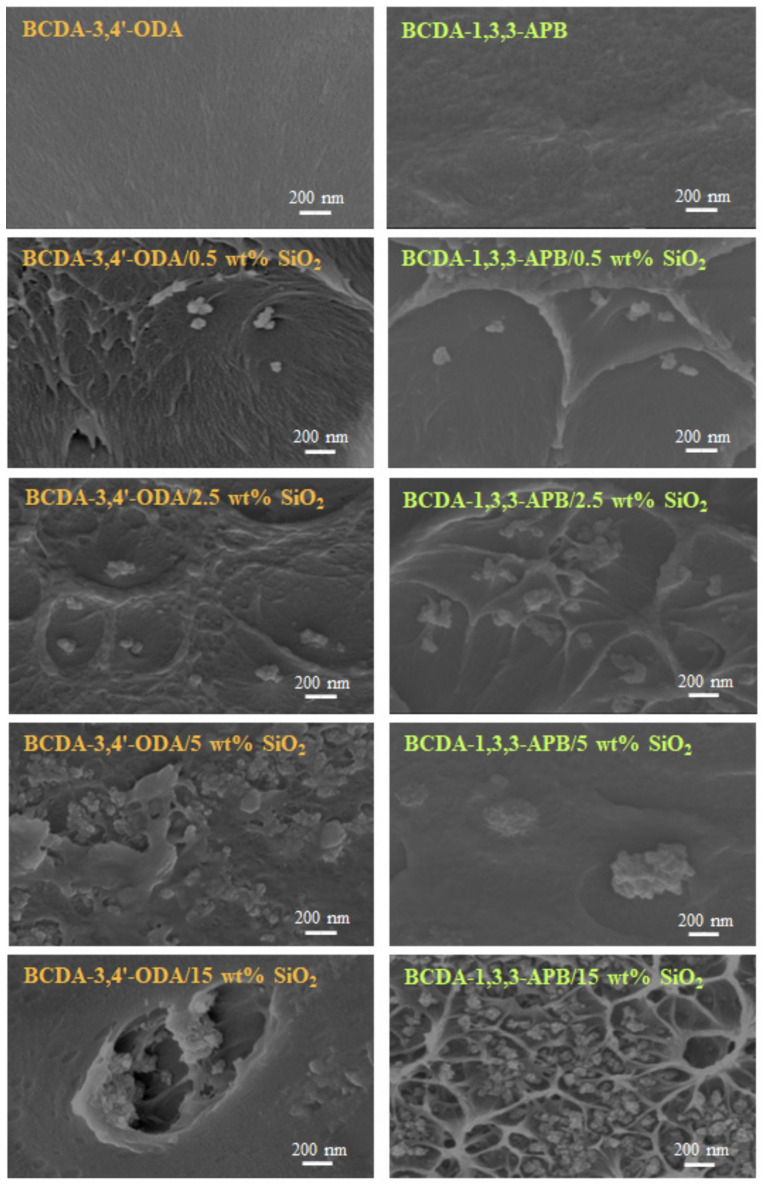
FE-SEM images of pristine alicyclic PI membranes and their related PI/SiO_2_ mixed matrix membranes.

The thickness for pristine PI membranes and PI/SiO_2_ MMMs ranged from 20 to 40 μm such that the average thickness was 30 μm. The membrane structures were observed via FE-SEM, and the cross-sectional images are displayed in Figure 5. When 0.5 wt% SiO_2_ was filled into the PI matrix, clusters of several SiO_2_ nanoparticles were created. This phenomenon could be attributed to the particle–particle interaction formed via hydrogen bonding [43,55]. With increasing SiO_2_ content, particle agglomeration became more and more serious. As the SiO_2_ content was raised to 15 wt%, large defects appeared due to the severe particle-cluster aggregation. A similar trend occurred in the literature with mesoporous SiO_2_ spheres (1.8–2 μm) dispersed into PVA membranes [56]. Because the membrane defects greatly deteriorated the pervaporation performance, the PI/15 wt% SiO_2_ MMMs were not suitable for pervaporation.

Table 2 presents the water contact angles of pristine alicyclic PI membranes and their related PI/SiO_2_ MMMs. The PI with BCDA-3,4′-ODA had a lower contact angle (about 10° lower) than that with BCDA-1,3,3-APB, indicating that BCDA-3,4′-ODA was more hydrophilic than BCDA-1,3,3-APB (with one more phenoxy group). However, the addition of SiO_2_ nanoparticles gave opposite effects on the water contact angle for both PIs. The water contact angle increased for the SiO_2_ incorporation on BCDA-3,4′-ODA/SiO_2_ MMMs, while the value decreased in the case of BCDA-1,3,3-APB/SiO_2_ MMMs. In most literatures incorporating SiO_2_ particles into dehydration pervaporation membranes [56,57,58,59], the water contact angle decreased with the increased SiO_2_ wt%. Our case of BCDA-1,3,3-APB/SiO_2_ MMMs exhibited a similar tendency, which implies that BCDA-1,3,3-APB/SiO_2_ MMMs became slightly more hydrophilic than the pristine BCDA-1,3,3-APB PI membrane [56,57,58,59]. On the contrary, the water contact angle for the composite coating via filling SiO_2_ nanoparticles into waterborne fluorine-containing epoxy was found to increase with the increased SiO_2_ content in the work of [60]; a superhydrophobic surface was created with the inclusion of fumed SiO_2_ nanoparticles inside a PDMS pervaporation membrane for ethanol recovery [61]. Similar to these results, the as-prepared BCDA-3,4′-ODA/SiO_2_ MMMs was a little less hydrophilic than the pristine BCDA-3,4′-ODA PI membrane.

### 3.2. Swelling Effects

The degree of swelling may be used as an indication of the affinity between polymer and a certain solvent [62]. The experimental results of pure alicyclic PI membranes on the degree of swelling for the three solvents are in the following order: for BCDA-3,4′-ODA, n-butanol (61%) > ethanol (57%) > water (47%); for BCDA-1,3,3-APB, n-butanol (55%) > ethanol (25%) > water (10%). As indicated in the literature [63] using aromatic PI membranes, the degree of swelling for 90 wt% aqueous ethanol solution was higher than that for 10 wt% aqueous ethanol solution; that is, the aromatic PIs exhibited a stronger affinity with ethanol molecules than water molecules. In [62], the pure membranes of commercial PIs such as Matrimid, Torlon, and P84 showed the solvent uptake ratio in an order of n-butanol > isopropanol > ethanol > water. Our results of alicyclic PI membranes had the same tendency on solvent affinity as theirs.

To theoretically investigate the membrane solubility, Hansen solubility parameters [64] are usually adopted. The total solubility parameter δ ((MJ/m^3^)^1/2^) includes three terms: dispersion (δ_d_), polarity (δ_p_), and hydrogen bonding (δ_h_):

(7) $$\delta^{2}=\delta_{d}^{2}+\delta_{p}^{2}+\delta_{h}^{2}.$$

The values of Hansen solubility parameters for common polymers could be found from the literature, but not for the polymers with more complex, uncommon, or newly synthesized monomers. In our case, the Van-Krevelen–Hoftyzer (VKH) method [65] was used to decompose the functional groups of alicyclic PI and then sum up the group contributions for evaluating the solubility parameter value, as follows:

(8) $$\delta_{d}=\sum \frac{F_{di}}{V},$$

(9) $$\delta_{p}=\frac{\sqrt{\sum F_{pi}^{2}}}{V},$$

(10) $$\delta_{h}=\sqrt{\frac{\sum E_{hi}}{V}},$$

(11) $$V=\frac{M}{\rho },$$

where F_di_: dispersion term (J^1/2^cm^3/2^mol^−^^1^); F_pi_: polar term (J^1/2^cm^3/2^mol^−^^1^); E_hi_: hydrogen bonding term (J/mol); V: molar volume (cm^3^/mol); ρ: density (g/cm^3^); M: molecular weight of the repeating unit (g/mol). In order to judge the affinity between solvent and polymer, δ_m,c_, the interaction force between polymer (m) and solvent (c) was calculated from the following equation [66,67,68]:

(12) $$\delta_{m,c}=\sqrt{{\left(\delta_{d,m}-\delta_{d,c}\right)}^{2}+{\left(\delta_{p,m}-\delta_{p,c}\right)}^{2}+{\left(\delta_{h,m}-\delta_{h,c}\right)}^{2}}.$$

The values of δ_d_, δ_p_, δ_h_, δ, and δ_m,c_ for alicyclic PIs, SiO_2_, and three solvents are presented in Table 3. In general, the smaller the δ_m,c_ value, the higher the affinity between polymer and solvent [66].

As shown in Table 3, BCDA-3,4′-ODA had slightly lower δ_m,c_ values for the three solvents than BCDA-1,3,3-APB. That is, BCDA-3,4′-ODA should have had slightly better affinities with the three solvents than BCDA-1,3,3-APB, which matched the results of solvent swelling effect. Moreover, the order of δ_m,c_ value for both PIs was butanol < ethanol < water. In reverse order, the affinity between alicyclic PI and solvent became butanol > ethanol > water. This sequence was consistent with the order of the solvent swelling degree obtained in this work. On the other hand, the Hansen solubility parameters of SiO_2_ and its related δ_m,c_ values for the three solvents were also evaluated, and the data are reported in Table 3. The order of δ_m,c_ value for SiO_2_ was butanol > ethanol > water, in an entirely opposite order to alicyclic PIs. These data suggest that SiO_2_ should be more hydrophilic than alicyclic PIs.

However, the results of water contact angle in Table 2 indicate that the incorporation of SiO_2_ nanoparticles in MMM exhibited the opposite tendency on hydrophilicity for a different PI matrix. Thus, the solvent solubilities of PI/SiO_2_ MMMs were investigated via measuring their solvent swelling degrees. The results are listed in Table 4. In the case of BCDA-3,4′-ODA/SiO_2_ MMMs, the increase in SiO_2_ wt% led to the decrease in the degree of swelling of pure water but an increase in pure butanol solubility. In contrast, an opposite trend was attained for BCDA-1,3,3-APB/SiO_2_ MMMs. These trends are in good agreement with the water contact angle results. Conclusively, the BCDA-3,4′-ODA/SiO_2_ MMMs became less hydrophilic due to the incorporation of SiO_2_ nanoparticles, whereas BCDA-1,3,3-APB/SiO_2_ MMMs were more hydrophilic with SiO_2_ loading. The phenomena on solvent swelling degrees displayed in the literature adopting chitosan/SiO_2_ xerogel MMMs [59] were similar to our BCDA-1,3,3-APB/SiO_2_ MMM case, since the tendency of water contact angle was the same.

### 3.3. Pervaporation Performance

**Figure 6:**
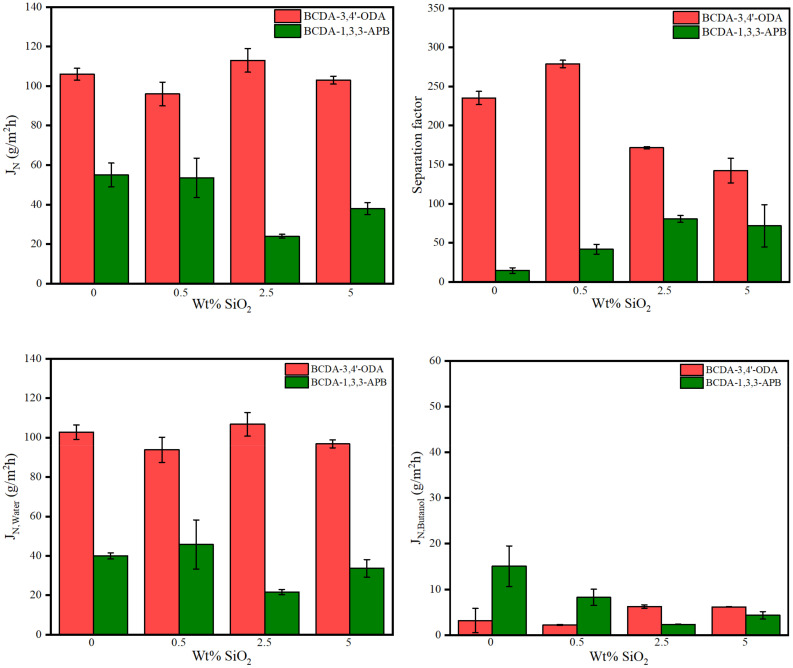
Pervaporation performance of pristine alicyclic PI membranes and their related PI/SiO_2_ mixed matrix membranes.

The pervaporation process of 85 wt% n-butanol aqueous solution was conducted at 40 °C using one piece of membrane disc (effective surface area: 19.63 cm^2^) in this study. The results of the total permeation flux and separation factor are illustrated in Figure 6. Due to the membrane thickness variation, the normalized permeation flux (J_N_) in terms of 30 μm (the average membrane thickness) was expressed. For pristine alicyclic PI membranes, BCDA-3,4′-ODA exhibited both a larger flux and a higher separation factor than BCDA-1,3,3-APB.

Worth reminding is that solution–diffusion is the principal separation mechanism for pervaporation through a dense membrane [14,15]. In the previous section discussing the swelling effect, BCDA-3,4′-ODA certainly revealed higher solvent affinities than BCDA-1,3,3-APB. To further understand the diffusion discrepancy for both alicyclic PIs, fractional free volume (FFV) was evaluated. FFV is usually applied to quantify the gap between polymer segments. As FFV increases, solvent molecules pass through the membrane more easily, resulting in an increased flux. The calculation of FFV is as follows [69]:

(13) $$FFV=\frac{V-V_{0}}{V}=1-\rho_{m}V_{0},$$

(14) $$V_{0}=1.3V_{W},$$

where V: specific volume of polymer (=1/ρ_m_) (cm^3^/mol); V_0_: occupied volume of polymer at 0 K (cm^3^/mol); ρ_m_: molar density of polymer (mol/cm^3^); V_W_: Van der Waals volume (cm^3^/mol). V_W_ value was estimated by Bondi’s model of group contribution [70]. The results verified that BCDA-3,4′-ODA had a larger FFV value (0.189) than BCDA-1,3,3-APB (0.171). More free volume allowed more solvent molecules to pass through the PI membrane with BCDA-3,4′-ODA. Since both the solvent solubility and diffusivity of BCDA-3,4′-ODA were higher than BCDA-1,3,3-APB, the resulting total permeation flux of BCDA-3,4′-ODA was two-fold that of BCDA-1,3,3-APB.

In addition, the individual fluxes of water and n-butanol were separately plotted in Figure 6. For both pristine PI membranes, the water flux was much larger than the butanol flux, such that the total flux was close to the individual water flux. This phenomenon is totally opposite to their swelling results in Table 4, where n-butanol could be uptaken and sorbed into the pure PI membrane more than water. The contradictory trend between solvent swelling degree and permeation flux for water/alcohol systems was also reported for the chitosan membrane [59] and BPDA-PI membrane [63]. The molecular dynamic diameter of water (2.7 Å) is smaller than butanol (5.5 Å) [59] so that water has a faster diffusion capability than butanol. Diffusion accelerated more water molecules across the dense PI membrane, resulting in a high water percentage at the permeate flux. Consequently, a large separation factor of water over butanol was yielded. The separation ability of our PI membranes was mainly accredited to the diffusion mechanism. The dominant diffusivity process of pervaporation was also found in the literature [58] using the PVA/nano silica composite membranes.

To evaluate the effect of SiO_2_ incorporation, the pervaporation results of the alicyclic PI/SiO_2_ MMMs are also presented in Figure 6. The tendency of total flux was close to that of individual water flux because the water content in permeate was very big. Moreover, both kinds of PI/SiO_2_ MMMs revealed similar effects on permeation fluxes, although they had opposite effects on membrane hydrophilicity (analyzed from the data of water contact angle and degree of swelling). The water flux values in most MMM cases were less than that of pristine PI membrane. On the other hand, with the increased SiO_2_ content, the n-butanol flux decreased first and then increased for both types of PI/SiO_2_ MMMs. The variation in solvent flux may be contributed from three effects: The first one is the degree of swelling [59], and a reminder that both types of PI/SiO_2_ MMMs exhibited the opposite tendency in solvent swelling degree. The second effect is the restriction on liquid transport due to the blockage of nonporous SiO_2_ nanoparticles [71], which would reduce the solvent flux. The third possible effect is the creation of non-selective transport paths owing to the void formation from SiO_2_ nanoparticle aggregation [43,56,58,59], which usually occurs at higher loading content. The combination of these phenomena led to different trends for water flux and n-butanol flux. Water flux may be interfered more with the addition of nonporous SiO_2_ nanoparticles. Since diffusion transport took over the permeation rate, this influence had even overwhelmed the effect of increasing water affinity for SiO_2_ loading in BCDA-1,3,3-APB/SiO_2_ MMMs. As for n-butanol flux, the combined effect resulted in the minimal flux appearing at 0.5 wt% SiO_2_ loading for BCDA-3,4′-ODA/SiO_2_ MMMs and 2.5 wt% for BCDA-1,3,3-APB/SiO_2_ MMMs.

In the aspect of the separation factor, both PI/SiO_2_ MMMs displayed a similar effect: the separation factor increased first and then decreased with the increasing filler loading. Similar trends were also indicated in the literatures [56,59]. In Figure 6, the optimal separation factor occurred at 0.5 wt% for BCDA-3,4′-ODA/SiO_2_ MMMs and 2.5 wt% for BCDA-1,3,3-APB/SiO_2_ MMMs, which were identical to the SiO_2_ content for the minimal n-butanol flux in each case. The separation factor was raised mainly by the decrease in n-butanol flux. In addition, the incorporation of SiO_2_ nanoparticles was more effective for the case of BCDA-1,3,3-APB/SiO_2_ MMMs, especially to enhance the separation factor at 3–5.7-fold.

**Figure 7:**
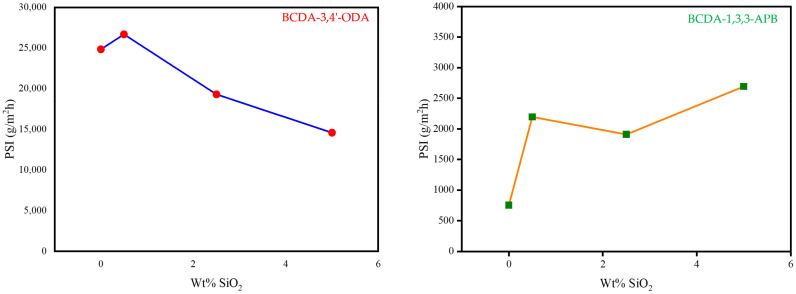
Variation in PSI (pervaporation separation index) with the increasing SiO_2_ wt% in PI/SiO_2_ mixed matrix membrane.

By combining the two important pervaporation outcomes (total flux and separation factor) together, the PSI value for each membrane was calculated as the index of separation efficiency. The PSI data are plotted in Figure 7. Based on the PSI performance, the optimal SiO_2_ content was 0.5 wt% for BCDA-3,4′-ODA/SiO_2_ MMMs and 5 wt% for BCDA-1,3,3-APB/SiO_2_ MMMs. In the case of BCDA-1,3,3-APB, the maximum PSI value did not occur at the maximal separation factor (2.5 wt% from Figure 6). The value of total permeation flux had a more significant contribution on the evaluation of PSI in this case, due to the fact that the separation factor values for BCDA-1,3,3-APB/SiO_2_ MMMs were not high. However, at the optimal filler content, the PSI value of BCDA-1,3,3-APB/5 wt% SiO_2_ MMM was raised 3.6-fold of pure PI membrane, while that of BCDA-3,4′-ODA/0.5 wt% SiO_2_ MMM was enhanced by only 8%.

The water/n-butanol pervaporation performances using the as-prepared alicyclic PI/SiO_2_ MMMs in this study are compared to those of other membranes reported in the literatures, as listed in Table 5. Note that the overall separation efficiency (PSI) of BCDA-3,4′-ODA-based membranes is about 10–30 times higher than that of BCDA-1,3,3-APB-based membranes. Only the PSI values of pristine alicyclic PI membrane and the PI/SiO_2_ MMM with optimal SiO_2_ content are presented in Table 5 for comparison. The PSI values of BCDA-1,3,3-APB-based membranes are only comparable to those of silica membranes, but they have better permeation flux than silica, pristine PBI, Torlon-based, P84-based, and PPSU-based membranes. On the other hand, the BCDA-3,4′-ODA-based membranes are superior to commercial PI/30% hPIM-1, PPSU-based, PVA cross-linked by citric acid, and silica membranes in both permeation flux and PSI. Consequently, large permeation flux is the chief feature of our alicyclic PI/SiO_2_ MMMs.

## 4. Conclusions

Two types of alicyclic PI/SiO_2_ MMMs were developed for water/n-butanol pervaporation. The results of water contact angle and pure solvent swelling degree on membrane characterization showed that the BCDA-3,4′-ODA/SiO_2_ MMMs became less hydrophilic with the increasing SiO_2_ content, while the BCDA-1,3,3-APB/SiO_2_ MMMs became more hydrophilic. In addition to the effect of the solvent swelling degree, the other effects affecting the pervaporation performance included: fractional free volume of pristine PI, faster diffusion capability of water, the restriction on liquid transport due to the blockage of nonporous SiO_2_ nanoparticles, and the creation of non-selective transport paths owing to the void formation from SiO_2_ nanoparticle aggregation at higher loading content. The combination of these effects resulted in the variations of individual solvent flux and separation factor. The BCDA-3,4′-ODA/SiO_2_ MMMs exhibited both higher permeation flux and greater separation factor than BCDA-1,3,3-APB/SiO_2_ MMMs. For both kinds of PI/SiO_2_ MMMs, the separation factor increased first and then decreased with the increasing SiO_2_ loading. The effect of SiO_2_ incorporation was more significant for the BCDA-1,3,3-APB/SiO_2_ MMMs, especially enhancing the separation factor 3–5.7 times. On the other hand, the overall separation efficiency (PSI value) of BCDA-3,4′-ODA-based membranes was better, ca. 10–30-fold higher than BCDA-1,3,3-APB-based membranes. Based on the PSI performance, the optimal SiO_2_ loading content was 0.5 wt% for BCDA-3,4′-ODA/SiO_2_ MMMs and 5 wt% for BCDA-1,3,3-APB/SiO_2_ MMMs. In addition, larger SiO_2_ content in MMM (e.g., 15 wt%) would cause more severe particle-cluster aggregation and create big defects to deteriorate the pervaporation performance.

## Figures and Tables

**Table 1:** Values of actual SiO_2_.wt% in alicyclic PI/SiO_2_ MMMs obtained from TGA.

SiO_2_ wt% Used in Experiment	0.5 wt%	2.5 wt%	5 wt%	15 wt%
BCDA-3,4′-ODA/SiO_2_ MMM	0.7 wt%	2.6 wt%	6.1 wt%	17.5 wt%
BCDA-1,3,3-APB/SiO_2_ MMM	0.6 wt%	3.0 wt%	7.1 wt%	17.4 wt%

**Table 2:** Water contact angles of pristine alicyclic PI membranes and their related PI/SiO_2_ mixed matrix membranes.

SiO_2_ wt% Used in Experiment	0 wt%	0.5 wt%	2.5 wt%	5 wt%	15 wt%
BCDA-3,4′-ODA/SiO_2_ MMM	64 ± 2°	64 ± 2°	65 ± 3°	70 ± 3°	72 ± 2°
BCDA-1,3,3-APB/SiO_2_ MMM	75 ± 1°	72 ± 1°	71 ± 2°	70 ± 1°	66 ± 2°

**Table 3:** Values of Hansen solubility parameters for alicyclic PIs, SiO_2_, and three solvents.

	$\delta_{D}$	$\delta_{P}$	$\delta_{H}$	δ
BCDA-3,4′-ODA	15.3	6.3	8.3	18.5
BCDA-1,3,3-APB	16.1	5.3	8.0	18.7
SiO_2_	18	27.5	29	43.8
Butanol	16	5.7	15.8	23.2
Ethanol	15.8	8.8	19.4	26.5
Water	15.5	16	42.3	47.8
$\delta_{m,c}$	**BCDA-3,4** **′** **-ODA**	**BCDA-1,3,3-APB**		**SiO_2_**
Butanol	7.6	7.8		25.6
Ethanol	11.4	11.9		21.1
Water	35.4	35.9		17.8

**Table 4:** Degrees of swelling for pristine alicyclic PI membranes and their related PI/SiO_2_ mixed matrix membranes.

SiO_2_ wt% Used in Experiment		0 wt%	0.5 wt%	2.5 wt%	5 wt%	15 wt%
BCDA-3,4′-ODA/SiO_2_ MMM	water	47%	48%	47%	44%	38%
	n-butanol	61%	69%	79%	83%	92%
BCDA-1,3,3-APB/SiO_2_ MMM	water	10%	19%	22%	24%	39%
	n-butanol	55%	48%	37%	34%	31%

**Table 5:** Comparison of water/n-butanol pervaporation performance of alicyclic PI/SiO_2_ mixed matrix membranes with literatures.

Membrane	Feed Conc. (wt%)	Temp. (°C)	Flux (g/m^2^h)	Separation Factor	PSI (g/m^2^h)	Ref.
BCDA-3,4′-ODA	85	40	106	235	24,804	This work
BCDA-3,4′-ODA/0.5 wt% SiO_2_	85	40	96	279	26,688	This work
BCDA-1,3,3-APB	85	40	55	15	770	This work
BCDA-1,3,3-APB/5 wt% SiO_2_	85	40	38	72	2698	This work
Matrimid/0% hPIM-1	85	60	24.8	5661	140,368	[63]
Matrimid/30% hPIM-1	85	60	109	72	7739	[63]
Torlon/0% hPIM-1	85	60	11.3	5661	63,958	[63]
Torlon/30% hPIM-1	85	60	30	655	19,620	[63]
P84/0% hPIM-1	85	60	18	5661	101,880	[63]
P84/30% hPIM-1	85	60	52.2	74	3811	[63]
PBI	85	60	11.6	>5000	>57,988	[35]
PBI/58%ZIF-8	85	60	226	698	157,522	[35]
CS/SiO_2_ xerogel (0.25 wt%)	90	25	476	1930	918,204	[59]
CS/SiO_2_ xerogel (0.25 wt%)	90	75	817	285	232,028	[59]
PPSU	85	60	28	395	11,032	[72]
5%-sPPSU	85	60	35	659	23,030	[72]
PVA cross-linked by citric acid	90	30	82	171	13,940	[73]
Silica membrane with α- and γ-alumina support layers	95	75	4.5	600	2696	[73]
Silica membrane with γ-alumina substrate tube	95	75	3	250	747	[73]

## Data Availability

Not applicable.
